# Review: Polymeric-Based 3D Printing for Tissue Engineering

**DOI:** 10.1007/s40846-015-0038-3

**Published:** 2015-06-10

**Authors:** Geng-Hsi Wu, Shan-hui Hsu

**Affiliations:** Institute of Polymer Science and Engineering, National Taiwan University, No. 1, Sec. 4 Roosevelt Road, Taipei, 10617 Taiwan, ROC; Research Center for Developmental Biology and Regenerative Medicine, National Taiwan University, Taipei, 100 Taiwan, ROC

**Keywords:** Additive manufacturing (AM), Tissue engineering, Scaffold

## Abstract

Three-dimensional (3D) printing, also referred to as additive manufacturing, is a technology that allows for customized fabrication through computer-aided design. 3D printing has many advantages in the fabrication of tissue engineering scaffolds, including fast fabrication, high precision, and customized production. Suitable scaffolds can be designed and custom-made based on medical images such as those obtained from computed tomography. Many 3D printing methods have been employed for tissue engineering. There are advantages and limitations for each method. Future areas of interest and progress are the development of new 3D printing platforms, scaffold design software, and materials for tissue engineering applications.

## Introduction

Three-dimensional (3D) printing is a commonly used term that is often considered synonymous with additive manufacturing. 3D printing has drawn a lot of public attention, especially for its use in medical research. Additive manufacturing refers to a group of techniques that can generate a model with reduced waste and higher energy efficiency compared to those of conventional fabrication methods. The ability to create a 3D structure in a green and sustainable way through the use of 3D printing has taken fabrication techniques to a new level.

Currently, 3D printing technology can be used for tissue regeneration purposes. In the past two decades, increasing attention has been given to tissue engineering. With tissue and organ regeneration, the hurdles of traditional therapeutic methods may be overcome by autologous transplantation. As these technologies gain acceptance, the shortage of donor organs or chronic rejection of transplants may no longer be a problem.

The goal of tissue engineering is to create tissue or organ replacement strategies. Scaffolds play an important role in tissue engineering. They serve as templates for cell adhesion and the recruitment of cells to infiltrate deep into a defect site. Moreover, scaffolds can provide mechanical supports during tissue regeneration. With biomimetic scaffolds, researchers attempt to create an environment close to the natural extracellular matrix (ECM) of that organ, in which cells could be guided to create a new tissue with appropriate function.

Conventional scaffold fabrication methods include solvent casting and particulate leaching [1, 2], fiber spinning [3], emulsion freeze drying [4], and phase separation [5]. Polymer-based scaffolds can then be acquired. These methods have been studied extensively [6–9]. Various polymers have been crafted into scaffolds using these methods and tested. Although conventional scaffold fabrication techniques have been improved, the physical properties of scaffolds fabricated by these methods still have limitations (i.e., controlling scaffold pore size, geometry, and porosity). Moreover, it is difficult to control the shape and dimension of scaffolds using these methods.

Unlike conventional scaffold fabrication techniques, which are highly process-dependent, additive manufacturing is design-dependent for scaffold fabrication. The size, geometry, and porosity can be precisely controlled during additive manufacturing to a patient’s specification. In addition, scaffolds made using additive manufacturing techniques are highly reproducible. More importantly, a custom-made scaffold with specified dimensions and geometry can be prepared. When applying a reasonable design, the cell–cell interaction and cell–ECM interaction can be manipulated. Scaffold design can be performed easily with computer-aided design [10]. By adjusting the parameters of manufacturing, tissue engineering scaffolds can be made to fit different purposes.

## Fused Deposition Modeling

**Fig. 1:**
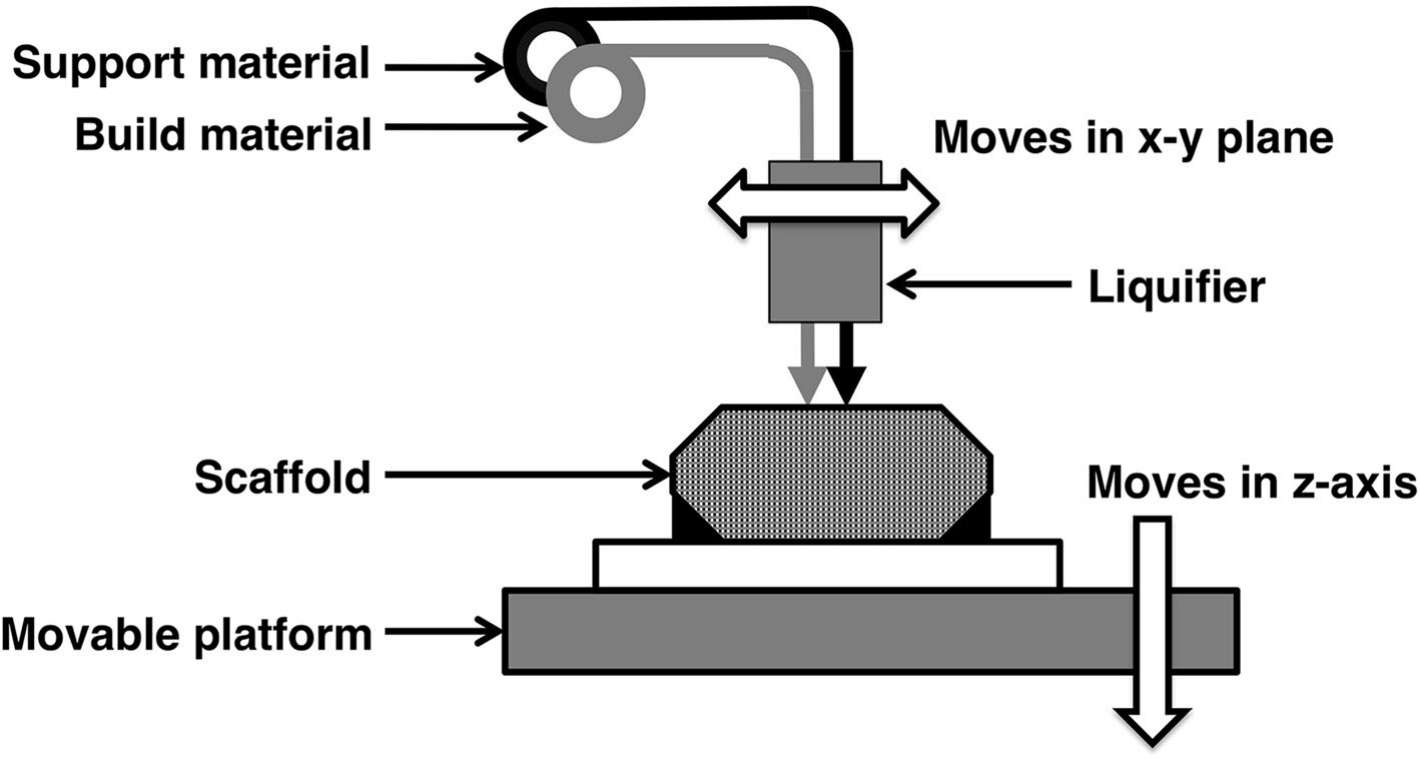
Scheme of fused deposition manufacturing (FDM). Melted polymer is extruded from nozzle to build scaffold

Various additive manufacturing techniques have been applied in tissue engineering. They can be categorized into two large groups according to the power source used during fabrication, namely heat or light. Fused deposition modeling (FDM) is a typical heat-using technique for 3D scaffold fabrication. A scheme of FDM is shown in Fig. 1. In this method, the filament of the desired material is fed and melted in a liquefier by heat before extrusion from the nozzle. The melted polymer is extruded from the nozzle and deposited layer by layer to create a scaffold. The process temperature depends on the melting temperature of building materials and is generally too high for cells to survive or for bioactive molecules to retain their activity. Zein et al. [11] fabricated a honeycomb-structured polycaprolactone (PCL) scaffold that has a channel size of 160–700 µm, a filament diameter of 260–370 µm, and a porosity of 48–77 %. The working temperature was determined as 125 ± 5 °C, which is considered a relatively narrow process window for polymer processing. Hsu et al. used poly(d,l-lactide) (PLA) as the feed material. Scaffolds with various fiber stacking orientations were produced and examined [12]. They also fabricated scaffolds with concentric cylinder geometry (with interconnected hollows) and tested them. Furthermore, collagen was placed in a poly(d,l-lactide-*co*-glycolide) (PLGA) scaffold to promote chondrocyte growth [13].

## Liquid Frozen Deposition Manufacturing

**Fig. 2:**
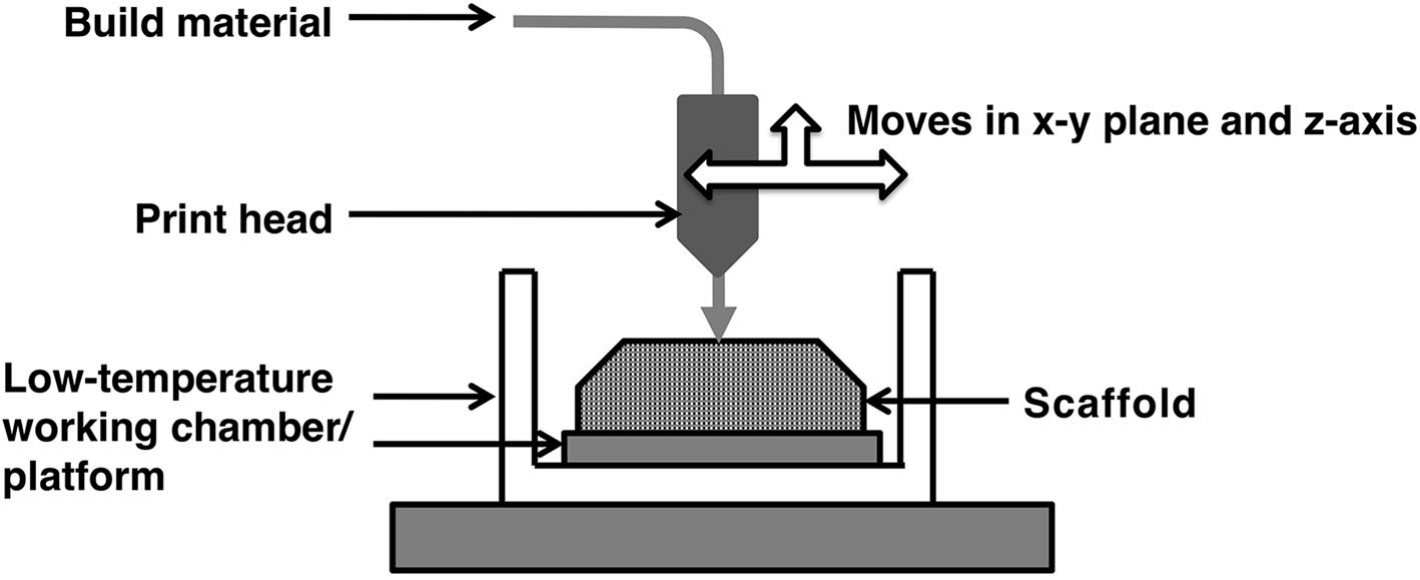
Scheme of liquid-frozen deposition manufacturing (LFDM). Low-temperature working chamber/platform is required in process

There are some drawbacks to FDM. During the process, the use of heat as the power source to melt the material can have undesired effects. The operating temperature of the system is too high for cells and other biomolecules. With this limitation, cells are hardly printed together with the material to form a cell-containing scaffold, and it is also difficult to incorporate biomolecules such as growth factors into the scaffold. To overcome the limitations associated with FDM, a lower-temperature cooling platform, called liquid frozen deposition manufacturing (LFDM), was developed. A scheme of LFDM is shown in Fig. 2. A low-temperature platform/chamber is required for the process.

LFDM involves low temperature during processing. Natural (e.g., chitosan) scaffolds as well as synthetic (e.g. PLGA) scaffolds were made with LFDM from polymer solutions by Hsu et al. for various applications [14–16]. Chitosan dissolved in acetic acid was printed and freeze-dried [14]. PLGA scaffolds were fabricated from a PLGA solution in an organic solvent (1,4-dioxane) using LFDM. The surface pore size of each of the stacking fibers was controlled by adjusting the concentration of the PLGA solution in the organic solvent. The pore size decreased from 2–3 µm to <1 µm as the concentration of PLGA solution was increased from 15 to 25 %. These scaffolds were found to promote the secretion of ECM from chondrocytes, which formed natural lacunae [15]. PLGA scaffolds were combined with alginate gel for the chondrogenesis of mesenchymal stem cells (MSCs) [16]. More recently, Hung et al. [17] developed a water-based system for printing polyurethane scaffolds. In their study, the organic solvent was replaced by water. Moreover, Xiong et al. [18] manufactured poly(l-lactic acid) (PLLA)/(tricalcium phosphate) composite scaffolds for bone tissue engineering. LFDM is considered as a more efficient procedure since it does not require heating. However, because LFDM normally requires freeze-drying after fabrication, it did not allow cells to be printed with the materials during the process. Although cells cannot be directly printed, it is expected that bioactive compounds or biomolecules could be incorporated with the scaffold during the process [19].

## Stereolithography

**Fig. 3:**
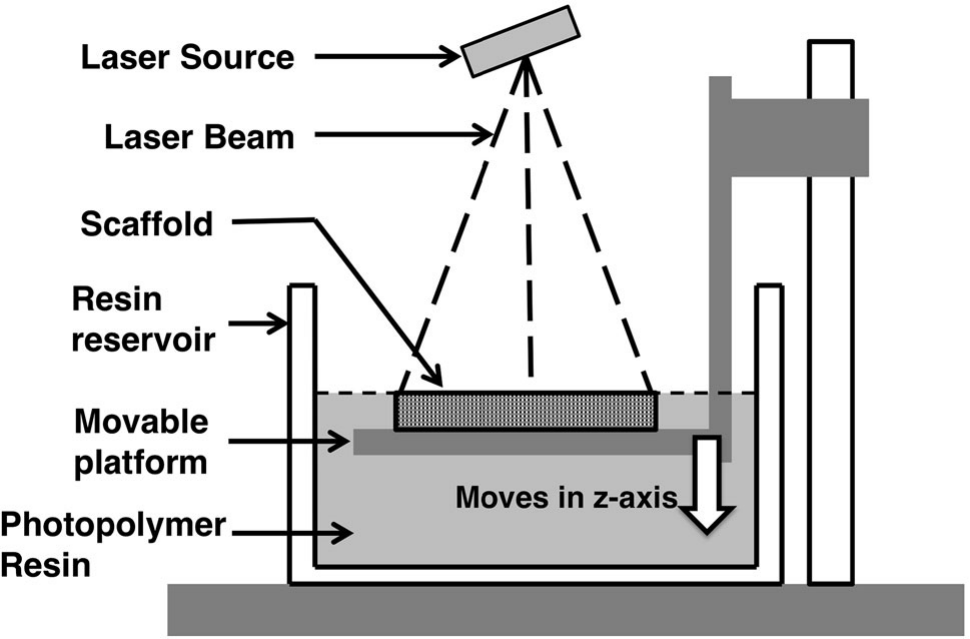
Scheme of stereolithography (SLA). Single laser beam scans surface of resin to polymerize or crosslink polymer resin

Stereolithography (SLA) employs a single beam laser to polymerize or crosslink a photopolymer resin. A scheme of SLA is shown in Fig. 3. By drawing on the liquid photopolymer resin with a light beam, thin layers of polymer are stacked layer by layer. A mixture of diethyl fumarate (DEF)/poly(propylene fumarate) (PFF) was used by Cooke et al. [20] to fabricate a scaffold. An 80-layer scaffold with a 4-mm thickness was fabricated using SLA. Holes and slots of various sizes were made on the scaffold. Protrusions were also made on the scaffold, which demonstrated the ability of SLA to build scaffolds various geometries. Melchels et al. [21] prepared a mathematically defined scaffold. The porous scaffold was built with two kinds of resin, either a PLA-based resin or a poly(d,l-lactide-*co*-ε-caprolactone)-based resin. By changing the pore size, resin selection, and pore architecture, the mechanical properties of the scaffold may be manipulated. Flexible and elastic materials could also be crafted into scaffolds via SLA. Schüller-Ravoo et al. used poly(trimethylene carbonate)-based resin to build scaffolds for cartilage tissue engineering [22]. When the scaffolds were seeded with bovine chondrocytes, glycosaminoglycans and fibrillar collagens were deposited after 6 weeks of culture. The resulting scaffolds presented a 50 % increase in compressive modulus.

In addition to stiff resin, hydrogels may be rendered as scaffolds through SLA processes [23–25]. While using hydrogel as the building material, the temperature is generally low enough for cells to survive. This makes it possible to encapsulate cells during scaffold fabrication. Dhariwala et al. [23] used a photopolymerizable hydrogel as the building material. Poly(ethylene oxide) (PEO) and poly(ethylene glycol) dimethacrylate were mixed and used as the building materials in the study. The resulting hydrogels did not have a high elastic modulus; however, the mechanical properties were comparable to those of other soft tissues (e.g., breast tissue). Furthermore, Chinese hamster ovary cells were successfully encapsulated in these hydrogel scaffolds. This result suggests that hydrogels may be used to encapsulate cells while maintaining cell viability. A PEO/poly(ethylene glycol) diacrylate (PEGDA) hydrogel was used to build scaffolds by Chan et al. [24]. The elastic moduli varied from 4.73 ± 0.46 to 503 ± 57 kPa, depending on the molecular weight of the PEGDA used in the hydrogels. With a wider range of elastic moduli, the hydrogels have more possibilities for various applications. NIH/3T3 cells have been encapsulated in hydrogel, retaining long-term viability. This was an important step for SLA in cell encapsulation. Seck et al. [25] produced a hydrogel structure with SLA using poly(ethylene glycol)/PDLA-based resins. Both porous and non-porous structures were prepared. The pore size of the porous structure ranged from 387 to 558 µm with an average size of 423 µm. Based on micro-computed tomography (µCT) data, a porosity of 52 % was determined, while the porosity of the designed architecture was 55 %. SLA processes have been used to render the internal and external morphology of scaffolds with high accuracy, and have the ability to build structures as designed. For a patient-specific tissue, Du et al. [26] constructed a viable artificial bone substitute with SLA through a series of manufacturing processes. With the use of µCT images, the constructs had the correct external shape and optimized internal channels.

## Digital Light Processing

**Fig. 4:**
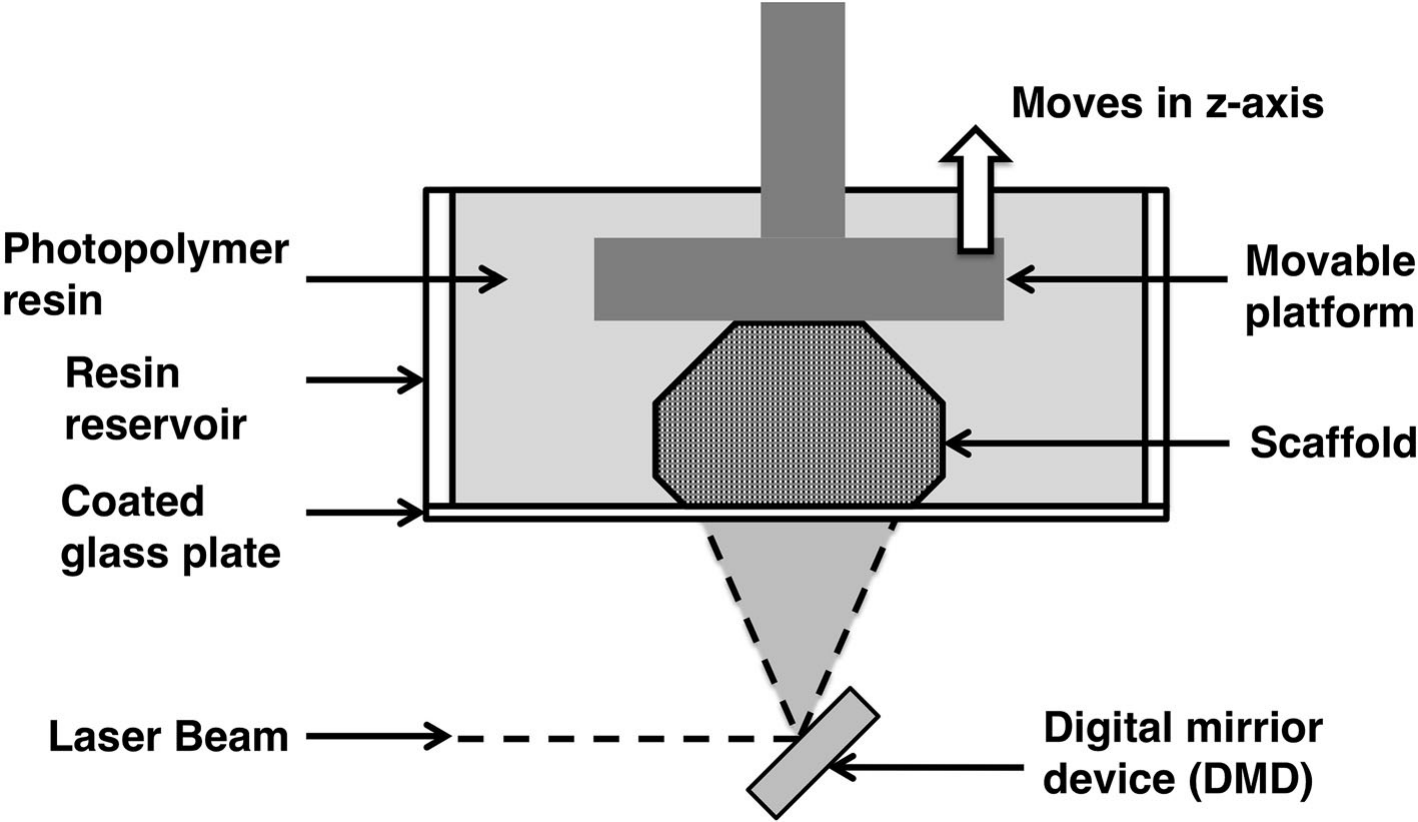
Scheme of digital light processing (DLP). Digital mirror device is used in process to illuminate entire layer of resin surface

Digital light processing (DLP) 3D printing uses a laser to cure a polymer. A scheme of DLP is shown in Fig. 4. Compared to SLA, which is a bottom-up process, DLP is a top-down process and is relatively faster. During the process, a digital mirror device (DMD) is used to control the curing laser beam. DMD has an array of micro-mirrors, which can rotate independently to control the laser beam to an on or off state. With the use of DMD, an entire layer can be cured at once, which makes DLP faster than the conventional SLA process. For tissue engineering, PEGDA hydrogel scaffolds were fabricated by Lu et al. [27] via DLP. In their study, murine-bone-marrow-derived cells were successfully encapsulated in the construct. A complex porous scaffold was fabricated by Gauvin et al. [28]. The hydrogel scaffold uses gelatin methacrylate (GelMA) as the building material. By varying the structure and the prepolymer concentration, the mechanical properties of scaffolds can be tuned. Furthermore, the interconnected pores allow for uniform distribution of human umbilical vein endothelial cells (HUVECs). As a result, scaffolds with high cell density and homogeneous cell distribution can be generated at the end of the culture period.

## Selective Laser Sintering

**Fig. 5:**
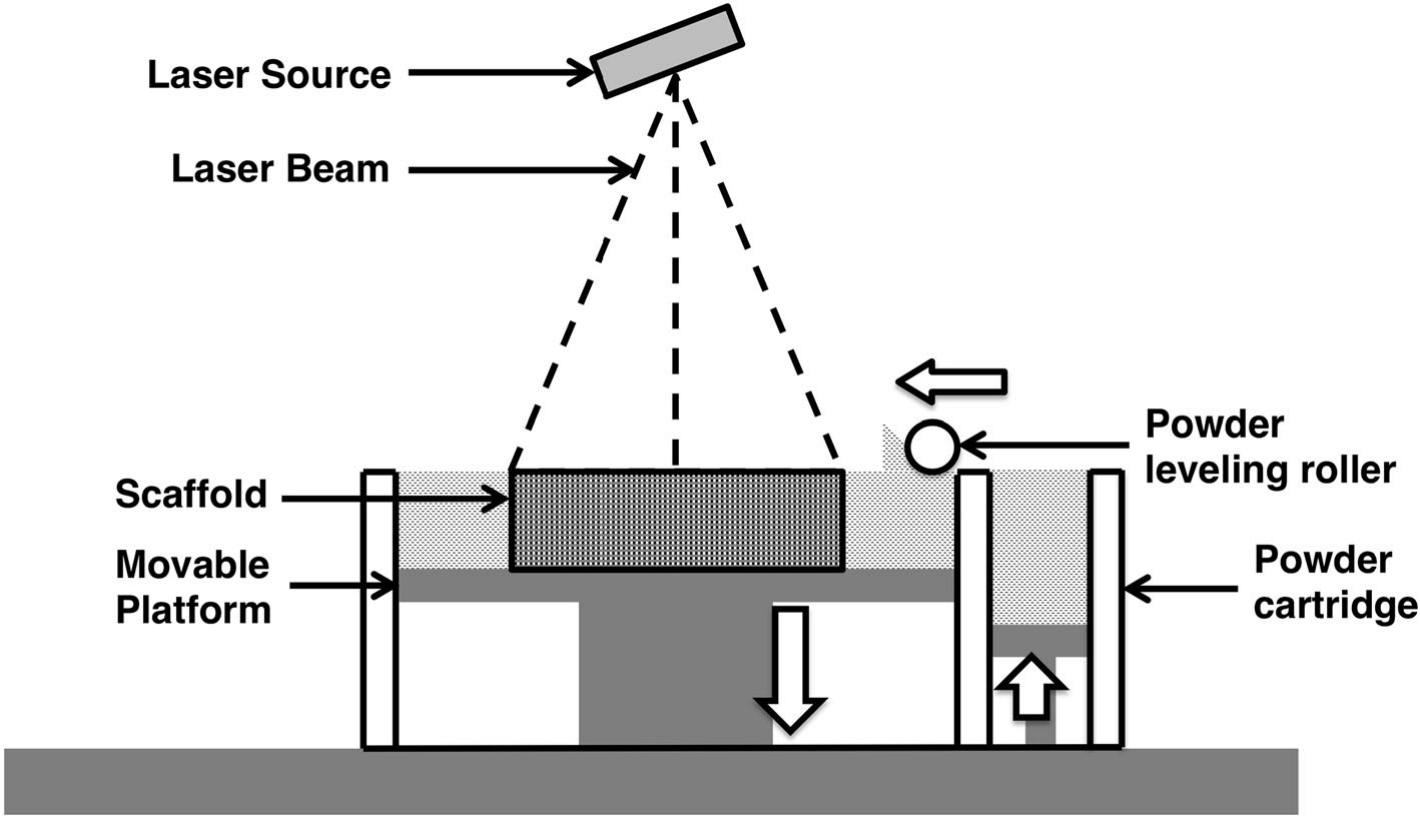
Scheme of selective laser sintering (SLS). Laser beam scans surface of polymer powder to sinter into scaffold

Selective laser sintering (SLS) is another technique commonly used in scaffold fabrication (as shown in Fig. 5). It uses a high-power laser for polymer powder sintering to form a scaffold. During sintering, a high-power laser, for example a carbon dioxide laser, is used to draw on the powders. The polymer powder can be fused into large parts, and thus the scaffold is made layer by layer. This technique is preferred for rendering complex porous scaffolds. Unlike FDM and SLA, SLS does not require supports. The unsintered powder provides support for the model during the build process. For bone tissue engineering, Williams et al. [29] manufactured porous PCL scaffolds via SLS. The mechanical properties of the resulting scaffolds are within the lower range of those of human trabecular bone. The porous structure provides spaces for tissue ingrowth as well as sufficient mechanical strength. PCL/hydroxyapatite, a biocomposite, was used to fabricate tissue engineering scaffolds by Wiria et al. [30]. A porous polyvinyl alcohol (PVA) scaffold was fabricated for bone tissue engineering by Shuai et al. via SLS [31]. The porous structure of the scaffold was controllable and totally interconnected. The porosity of the scaffolds was measured to be 67.9 ± 2.7 %. A porous scaffold proposed by Yeong et al. [32] was fabricated for cardiac tissue engineering. In this study, SLS was used to fabricate PCL scaffolds. Both PLA and PCL scaffolds fabricated by SLS have demonstrated feasibility for specific tissue engineering applications. Chen et al. rendered PCL scaffolds for use in cartilage tissue engineering research [33]. Chondrocytes were seeded in collagen and further loaded into the scaffold. Studies on pore geometry and distribution were performed. Results showed that a customized and designed scaffold could be made with the combination of these technologies for cartilage tissue engineering. Regarding the starting materials for the SLS process, Ca-P/poly(hydroxybutyrate-*co*-hydroxyvalerate) nanocomposite material was used by Duan and Wang to fabricate microspheres [34]. Normally, bioactive molecules are not able to retain their activity after the SLS process. These microspheres could encapsulate proteins and are suitable for SLS processes to build up scaffolds for tissue regeneration. Although the encapsulation efficiency was only 24.51 ± 0.60 %, this study demonstrated the potential of biomolecule incorporation within the materials used for SLS scaffold fabrication. A summary of the advantages and disadvantages of various 3D printing techniques is shown in Table 1.

**Table 1:** Advantages and disadvantages of various 3D printing techniques

	Advantages	Disadvantages
Fused deposition modeling	Good mechanical properties; solvent not required	High temperature; filament required; narrow process window
Liquid-frozen deposition manufacturing	Low temperature; can incorporate biomolecules	Freeze-drying required
Low-temperature deposition manufacturing		
Stereolithography	Smoother surface; high resolution; fast processing	High cost; possibly high temperature; toxic uncured resin
Digital light processing	High resolution; fast processing; less shrinkage	High cost; toxic uncured resin
Selective laser sintering	No supports needed during manufacturing; high resolution; fast processing	Rough surface finish; high temperature
3D bioplotter	Cells and hydrogels can be printed	Low mechanical strength; slow processing; low accuracy

## Other Techniques

There are still many techniques in the field of additive manufacturing that remain to be explored for their use in tissue engineering. Compared to the techniques introduced above, some methods have higher resolution, allowing smaller line widths of the fabricated scaffold. Some processes are suitable for “printing” a scaffold and cells at the same time or for printing cells directly as materials, which are fused layer upon layer during scaffold rendering. With these kinds of techniques, cell-containing scaffolds can be fabricated. Kolesky et al. [35] printed perfusable channels, 45–500 µm in diameter, with a custom-built 3D printer (ABG 10000, Aerotech Inc., Pittsburgh, PA). They used cell-laden GelMA and pluronic F127 to print a heterogeneous tissue construct with perfusable channels as vasculature. Billiet et al. [36] fabricated hydrogel scaffolds using a bioplotter (EnvisionTEC, Gladbeck, Germany). The cells were printed with the scaffold during the process. A scaffold-free system was introduced by Norotte et al. with the use of a bioprinter, which was manufactured in-house [37]. Multicellular spheroids and cellular cylinders were used as the building blocks to leave channels for vascular tissue engineering.

## Challenges and Prospects

Additive manufacturing has a lot of advantages, but it still has many challenges that remain to be overcome. Firstly, the materials used in additive manufacturing are limited to the materials required for each technique. Few materials can be used in more than one 3D printing modality. Incorporating bioactive molecules is another challenge for additive manufacturing. Bioactive molecules may be sensitive to the printing environment. If the printing processes involve a solvent or extreme temperature, the folding of proteins can be affected or the proteins can be denatured. Methods suitable for bioactive molecule incorporation in 3D implants are limited. Moreover, the biocompatibility of the scaffold following successful but novel fabrication techniques must be evaluated. Given the limited number of commercially available materials, it may be challenging to control degradation, mechanical properties, pore size, and surface properties. These topics are discussed below.

Control of degradation rate is important for scaffolds used for tissue regeneration. The degradation rate should be tuned carefully to synchronize with the regeneration rate of the neotissue. For instance, poly(α-hydroxy esters) have been used to create scaffolds for a variety of biomedical applications [1, 3–5, 7, 38–40]. The degradation rate of these scaffolds strongly depends on the size and geometry of the product [38, 39]. The resolution of additive manufacturing techniques varies. Overall porosity and pore interconnectivity also affect the degradation rate [40]. When designing a scaffold, these parameters should be taken into consideration. Scaffold degradation byproducts have been studied for most of the materials used in tissue engineering. Most have good biocompatibility. However, fast-degrading polymers may cause an inflammatory response in vivo. Based on the degradation profile and degradation byproducts, the biocompatibility of materials should be evaluated as part of the design of the scaffold.

Since the function of a scaffold is to provide a biomimetic environment for cell attachment, proliferation and extracellular matrix secretion, suitable mechanical properties (e.g., similar to those of natural tissue) are important for 3D printed scaffolds. This would help cells maintain their phenotype and could induce the correct matrix secretion for the neotissue. Huang et al. designed a 3D environment for the maintenance of the spheroid morphology of MSCs [41]. It should be noted that 3D printing can sometimes produce scaffolds that are stiffer than those that can be fabricated using conventional methods. Although hydrogels are used to fabricate scaffolds, their mechanical strength may be insufficient. To improve the strength of hydrogel scaffolds, Wüst et al. developed a special hydrogel composite [42]. They used a two-step gelation process to make a mixture of alginate and gelatin hydrogel. Furthermore, hydroxyapatite was added to the hydrogel at various ratios to provide a mechanically tunable construct.

In addition to the mechanical properties, the microenvironment varies with tissue. Scaffold pore size requirements vary between different tissues and organs. In conventional scaffold fabrication, the control of the pore size strongly depends on the process [5, 43–45]. For instance, scaffolds fabricated from solution freeze-drying depend on the concentration of the solution and the size of the ice crystal [43, 44]. The advantage of additive manufacturing is the ability to accurately and precisely control the pore size and geometry [11, 13, 21, 25, 31, 32]. By adjusting fabrication parameters, various pore sizes may be easily achieved. However, with pore size control being possible, it is equally important to determine the optimal pore size needed for the regenerative process. New studies on pore size are needed.

Surface properties are another critical parameter for tissue engineering scaffolds. Surface properties include topography, hydrophobicity, and roughness. These surface features are important in cell–scaffold interactions as they affect how cells respond to the scaffold. For example, the surface of the scaffold from an SLS process is usually excessively rough. Although a rougher surface may increase cell attachment, overly sharp features may damage cells. The surface of a scaffold fabricated using the FDM method may be smooth and more suitable for cells. Yen et al. demonstrated that LFDM scaffolds with a rough surface (1–2 µm pores) may benefit the proliferation of attached chondrocytes [15]. The hydrophobicity of the scaffold may affect cell adhesion to the surface. Hsu et al. demonstrated that LFDM fabrication of PLGA scaffolds did not facilitate MSC seeding unless cells were embedded in alginate [16]. Hsu et al. fabricated chitosan scaffolds treated with air plasma [14], which reduced hydrophobicity and thereby enhanced cell seeding. After plasma treatment, the hydrophobicity of the scaffold was reduced, allowing cells to be seeded in the scaffolds more easily.

Finally, direct organ fabrication is the ultimate goal of additive manufacturing in tissue engineering. There is a possibility of printing a complete organ that could be directly transplanted into the human body. In this situation, the patterning of cells and materials in a printed scaffold would need to be carefully designed.

In conclusion, recent developments in tissue engineering include various new approaches for creating 3D scaffolds. Compared to conventional fabrication methods, additive methods allow scaffolds to be made quickly and accurately. Moreover, this technology could lead to custom-made scaffolds for patients. Further developments in additive manufacturing in tissue engineering will require new biomaterials, scaffold design optimization, and better knowledge of cell and organ physiology.
